# MiR-148a-3p within HucMSC-Derived Extracellular Vesicles Suppresses Hsp90b1 to Prevent Fibroblast Collagen Synthesis and Secretion in Silica-Induced Pulmonary Fibrosis

**DOI:** 10.3390/ijms241914477

**Published:** 2023-09-23

**Authors:** Qiyue Jiang, Jing Zhao, Qiyue Jia, Hongwei Wang, Wenming Xue, Fuao Ning, Jiaxin Wang, Yan Wang, Zhonghui Zhu, Lin Tian

**Affiliations:** 1 Department of Occupational and Environmental Health, School of Public Health, Capital Medical University, Beijing 100069, China; 2 Beijing Key Laboratory of Environmental Toxicology, Capital Medical University, Beijing 100069, China

**Keywords:** silica, pulmonary fibrosis, extracellular vesicles, HucMSCs, fibroblasts, MiR-148a-3p, Hsp90b1

## Abstract

Silicosis is a fatal occupational respiratory disease caused by the prolonged inhalation of respirable silica. The core event of silicosis is the heightened activity of fibroblasts, which excessively synthesize extracellular matrix (ECM) proteins. Our previous studies have highlighted that human umbilical cord mesenchymal stem cell-derived extracellular vesicles (hucMSC-EVs) hold promise in mitigating silicosis and the significant role played by microRNAs (miRNAs) in this process. Delving deeper into this mechanism, we found that miR-148a-3p was the most abundant miRNA of the differential miRNAs in hucMSC-EVs, with the gene heat shock protein 90 beta family member 1 (Hsp90b1) as a potential target. Notably, miR-148a-3p’s expression was downregulated during the progression of silica-induced pulmonary fibrosis both in vitro and in vivo, but was restored after hucMSC-EVs treatment (*p* < 0.05). Introducing miR-148a-3p mimics effectively hindered the collagen synthesis and secretion of fibroblasts induced by transforming growth factor-β1 (TGF-β1) (*p* < 0.05). Confirming our hypothesis, Hsp90b1 was indeed targeted by miR-148a-3p, with significantly reduced collagen activity in TGF-β1-treated fibroblasts upon Hsp90b1 inhibition (*p* < 0.05). Collectively, our findings provide compelling evidence that links miR-148a-3p present in hucMSC-EVs with the amelioration of silicosis, suggesting its therapeutic potential by specifically targeting Hsp90b1, thereby inhibiting fibroblast collagen activities. This study sheds light on the role of miR-148a-3p in hucMSC-EVs, opening avenues for innovative therapeutic interventions targeting molecular pathways in pulmonary fibrosis.

## 1. Introduction

Silicosis, an enduring and lethal fibrotic lung disease, arises from the long-term inhalation of free silica dust, leading to irreversible and life-threatening fibrotic lung damage. Regrettably, silicosis stands as one of the most serious and lethal occupational diseases in the world [1,2], with no effective way of reversing its clinical progression being established until now. Silicosis is distinguished by the formation of silicon nodules and diffuse interstitial fibrosis, primarily stemming from the excessive deposition of extracellular matrix (ECM) proteins such as collagen and fibronectin [3]. Myofibroblasts, known as activated fibroblasts that undergo proliferation and migration to the site of injury, are considered the primary contributor to the formation of the ECM [4]. Compared with the resting tissue-resident fibroblasts, activated fibroblasts have distinct characteristics, such as the excessive synthesis and secretion of ECM proteins and upregulation of alpha-smooth muscle actin (α-SMA), which is a myofibroblast marker. Activated fibroblasts also have contractile properties and are involved in ECM remodeling processes that contribute to the progression of pulmonary fibrosis [5,6]. The activation of fibroblasts driven by diverse stimulating factors represents a central event in the development of silica-induced pulmonary fibrosis. Consequently, targeting and impeding this process could hinder the progression of silica-induced pulmonary fibrosis [7,8].

Recently, mesenchymal stem cells (MSCs) have emerged as a compelling cellular therapy due to their remarkable regenerative capabilities. As a versatile treatment approach, MSCs hold great potential in facilitating the repair and regeneration of damaged lung tissue [9,10,11]. Many experimental studies have proved that the paracrine function of MSCs plays an indispensable role in the process of tissue repair [12]. Extracellular vesicles (EVs) are one of the main components of paracrine of MSCs [13]. Functioning as nanoscale membranous vesicles, EVs are actively released by cells and play a pivotal role in intercellular material exchange, information communication, and targeted regulation [14]. EVs mirror the biological function of the maternal cells in the treatment of diseases and offer a viable alternative to cell transplantation therapy, effectively circumventing the limitations associated with abnormal differentiation, embolism, and immunocompatibility [15]. However, the specific mechanisms underlying the therapeutic actions of EVs are yet to be fully comprehended.

MicroRNAs (miRNAs) are small and short single-stranded noncoding RNAs that regulate gene expression after transcription by binding to the 3′ untranslated region (3′ UTR) of the target mRNA sequence [16,17]. Multiple studies have shown that mesenchymal stem cell-derived extracellular vesicles (MSC-EVs) have a protective effect on pulmonary fibrosis, which is closely related to miRNAs [18,19]. In our prior study, we observed that specific miRNAs, such as let-7i-5p and miR-26a-5p, which originate from human umbilical cord mesenchymal stem cell-derived extracellular vesicles (hucMSC-EVs), were instrumental in inhibiting silica-induced pulmonary fibrosis in mice [20,21]. Through further research, we found that miR-148a-3p exhibited the highest abundance among the differentially expressed miRNAs in hucMSC-EVs. However, its role in silica-induced pulmonary fibrosis remains unknown. Studies have shown that miR-148a-3p may have an anti-fibrotic effect in liver fibrosis [22,23], tuberculous fibrosis [24], and idiopathic pulmonary fibrosis [19]. This has prompted us to investigate whether miR-148a-3p plays a protective effect in silica-induced pulmonary fibrosis. Meanwhile, within the scope of predicted target genes for miR-148a-3p, heat shock protein 90 beta family member 1 (Hsp90b1), also known as Grp94, is a member of the heat shock protein 90 (Hsp90) family [25,26]. Studies have shown that Hsp90b1 is highly expressed in hepatic fibrosis [27,28]; however, the specific role of Hsp90b1 in the context of silicosis remains unverified and warrants further investigation.

Thus, in this study, we investigate the impact of hucMSC-EVs on the collagen synthesis and secretion of fibroblasts, specifically focusing on the influence of miR-148a-3p present within hucMSC-EVs and its potential target gene Hsp90b1. This investigation involved in vitro experiments and in vivo assessments that were conducted utilizing a well-established silica-induced pulmonary fibrosis mice model.

## 2. Results

### 2.1. Cultivation of hucMSCs and Identification of hucMSC-EVs

**Figure 1:**
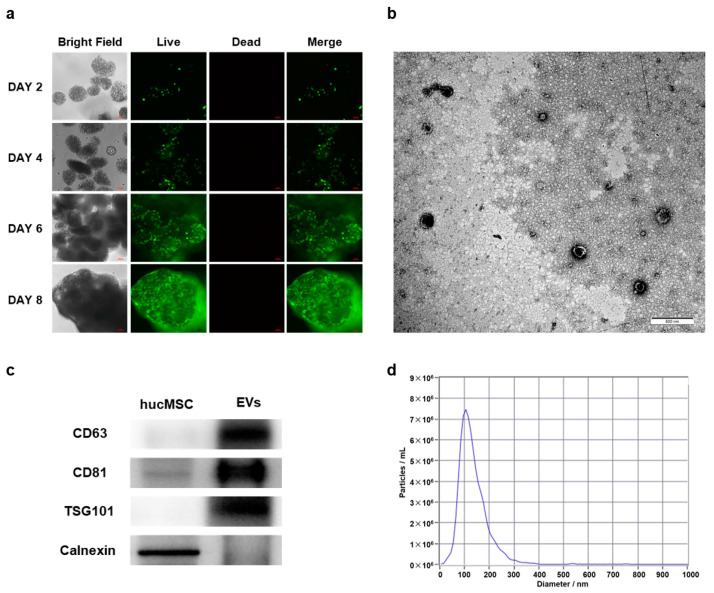
Cultivation of human umbilical cord mesenchymal stem cells (hucMSCs) and identification of human umbilical cord mesenchymal stem cell-derived extracellular vesicles (hucMSC-EVs). (**a**) Three-dimensional (3D) cultivation of hucMSCs on day 2, day 4, day 6, and day 8, as indicated by live (green) and dead (red) cell staining. Scale bar: 100 μm. (**b**) Transmission Electron Microscopy (TEM) image displaying the morphology of hucMSC-EVs. Scale bar: 500 nm. (**c**) Western blotting analysis of hucMSC-EVs biomarkers (CD63, CD81, TSG101, and Calnexin), with EVs representing hucMSC-EVs in the image. (**d**) Size distributions of hucMSC-EVs determined via Nanoparticle Tracking Analysis (NTA), revealing an average size of 133.5 nm.

In order to obtain a sufficient cell supernatant to extract hucMSC-EVs (using EVs to represent hucMSC-EVs in the image), a three-dimensional (3D) dynamic culture bioreactor system was employed to facilitate the robust expansion of hucMSCs. Figure 1a shows that the density of hucMSCs increased during continuous cell culture. Subsequently, the extracted hucMSC-EVs were identified via Transmission Electron Microscopy (TEM), Western blotting analysis, and Nanoparticle Tracking Analysis (NTA). Our results showed that hucMSC-EVs presented a sphere-shaped morphology (Figure 1b); the positive markers of EVs, including CD63, CD81, and TSG101, were intensively expressed in hucMSC-EVs when compared to the corresponding cells. In contrast, the negative marker Calnexin was detected in hucMSCs but notably absent in hucMSC-EVs (Figure 1c). Additionally, the average particle size of hucMSC-EVs was 133.5 nm, as illustrated in Figure 1d. These comprehensive findings collectively supported the classification of the hucMSC-EVs employed in this study as authentic extracellular vesicles, in accordance with established EV definitions and characteristics.

### 2.2. MiR-148a-3p Was Highly Expressed in hucMSC-EVs and Downregulated in Silica-Induced Pulmonary Fibrosis In Vitro and In Vivo

**Figure 2:**
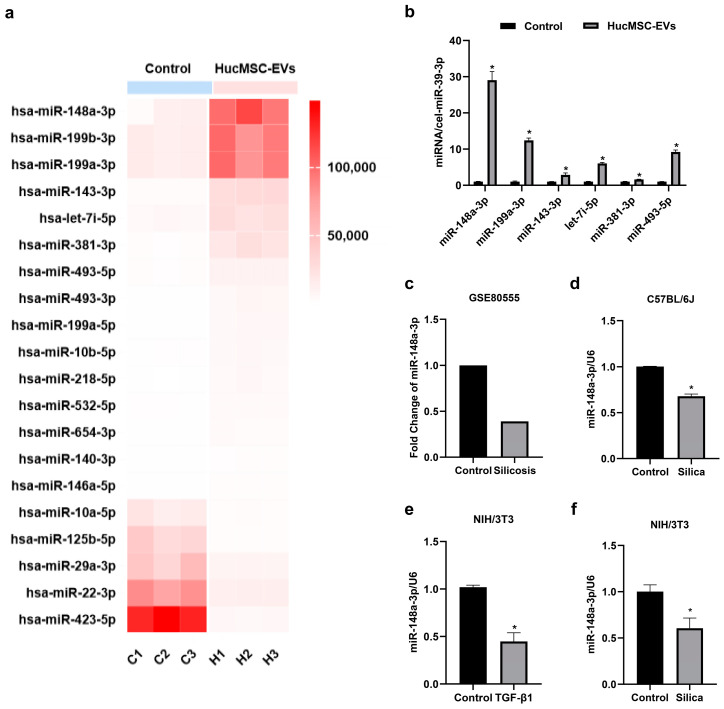
MiR-148a-3p was highly expressed in hucMSC-EVs and downregulated in the silica-induced pulmonary fibrosis in vitro and in vivo. (**a**) Heat map of differential microRNA (miRNA) expression in hucMSC-EVs. (**b**) Validation of miRNA-sequence results using quantitative real-time polymerase chain reaction (qRT-PCR). (**c**) Expression changes regarding miR-148a-3p in three healthy controls and three silicosis patients from a silicosis database (GSE80555). (**d**) Relative expression of miR-148a-3p in silica-induced mouse fibrotic lung tissues. (**e**) Relative expression of miR-148a-3p in transforming growth factor-β1 (TGF-β1)-stimulated NIH/3T3 cells. (**f**) Relative expression of miR-148a-3p in NIH/3T3 cells cultured in supernatants from silica-treated macrophages. * *p* < 0.05 compared with the control group. Data are shown as the means ± SD (n ≥ 3).

EVs could exert biological functions to regulate many fibrotic diseases, and miRNA is one of the essential components and effectors [29]. We analyzed miRNA expression in hucMSC-EVs via miRNA sequencing with the human embryonic lung fibroblast-derived extracellular vesicles (MRC-5-EVs) as the control group (Figure 2a). The accuracy of the miRNA sequencing results was further verified via qRT-PCR analysis (Figure 2b). Notably, we observed that miR-148a-3p exhibited the highest abundance among the differentially expressed miRNAs in hucMSC-EVs when compared with the control group (|Log2 (Fold Change)| > 2, *p* < 0.05), strongly indicating a potential association between miR-148a-3p and the functional role of hucMSC-EVs. Additionally, the involvement of various miRNAs in the development and progression of pulmonary fibrosis, as well as silica-induced pulmonary fibrosis, has been extensively reported in the literature [30,31]. In support of our findings, an analysis of a silicosis database (GSE80555) demonstrated that miR-148a-3p was downregulated in silicosis patients (Figure 2c). We observed a similar downregulation of miR-148a-3p in silica-exposed mouse lung tissue (Figure 2d). Furthermore, our in vitro investigations revealed a consistent reduction in miR-148a-3p levels. This reduction was observed not only in NIH/3T3 cells cultured in supernatants from silica-treated macrophages but also in cells stimulated with transforming growth factor-β1 (TGF-β1) (Figure 2e,f). These experimental results indicated that miR-148a-3p could be associated with silica-induced lung fibrosis processes in vitro and in vivo. Based on these results, we selected miR-148a-3p as the target miRNA of interest and focused on its role in hucMSC-EVs antagonism against silica-induced pulmonary fibrosis.

### 2.3. MiR-148a-3p Related to hucMSC-EVs’ Inhibition of Silica-Induced Pulmonary Fibrosis in Mice

**Figure 3:**
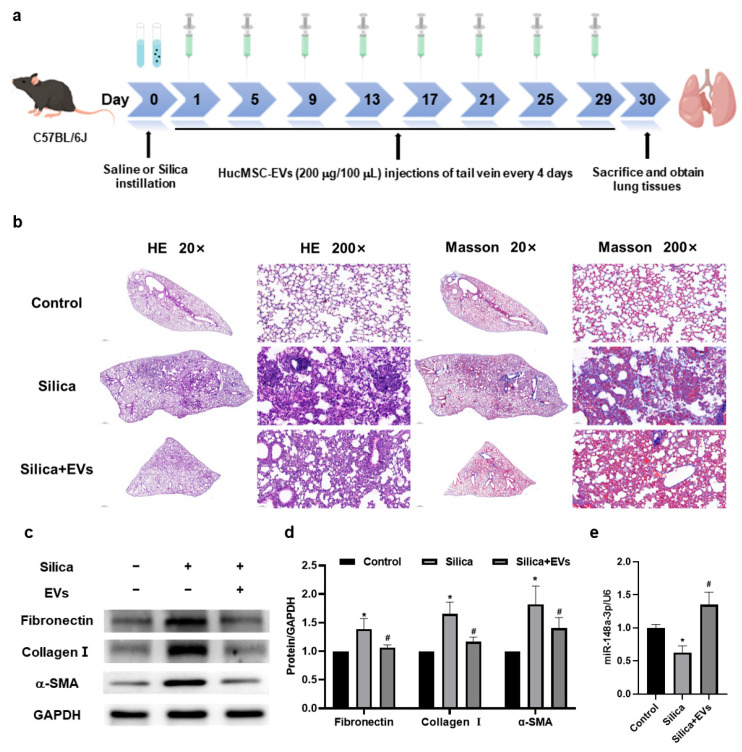
MiR-148a-3p related to hucMSC-EVs’ inhibition of silica-induced pulmonary fibrosis in mice. (**a**) The experimental procedure illustrating the administration of hucMSC-EVs (200 μg/100 μL) every 4 days following the instillation of saline or silica in mice. (**b**) Representative images of lung sections subjected to hematoxylin and eosin (HE) staining and Masson staining (magnifications: 20× and 200×). (**c**,**d**) Western blotting analysis showing the levels of Fibronectin, Collagen I, and alpha-smooth muscle actin (α-SMA) in the lung tissues of mice from three different groups. (**e**) Quantification of miR-148a-3p expression measured via qRT-PCR. * *p* < 0.05 compared with the control group; # *p* < 0.05 compared with the silica group. Data are shown as the means ± SD (n ≥ 3).

In order to verify our hypothesis in vivo, we performed a study using a mouse model of silica-induced pulmonary fibrosis (Figure 3a). Histological analysis with hematoxylin and eosin (HE) staining showed that, in contrast to the control group, the silica group exhibited a disrupted alveolar structure, thickened alveolar walls, compressed alveolar spaces, the infiltration of inflammatory cells, and the presence of varying-sized cell nodules in lung tissues. However, these changes were mitigated upon intervention by the hucMSC-EVs (Figure 3b). Masson staining demonstrated increased collagen deposition around the bronchioles, small blood vessels, and cell nodules in the silica group compared to the control group, which was attenuated following treatment with hucMSC-EVs. Additionally, Western blotting analysis revealed elevated protein expressions of Fibronectin, Collagen I, and α-SMA in the silica group, which were significantly reduced after hucMSC-EVs treatment (Figure 3c,d). Additionally, miR-148a-3p expression was found to be downregulated in the silica group but restored upon hucMSC-EV intervention (Figure 3e). The above results suggested that miR-148a-3p might relate to hucMSC-EVs’ inhibition of silica-induced pulmonary fibrosis in mice. 

### 2.4. MiR-148a-3p in hucMSC-EVs Suppressed TGF-β1-Induced Collagen Synthesis and Secretion in NIH/3T3 Cells

**Figure 4:**
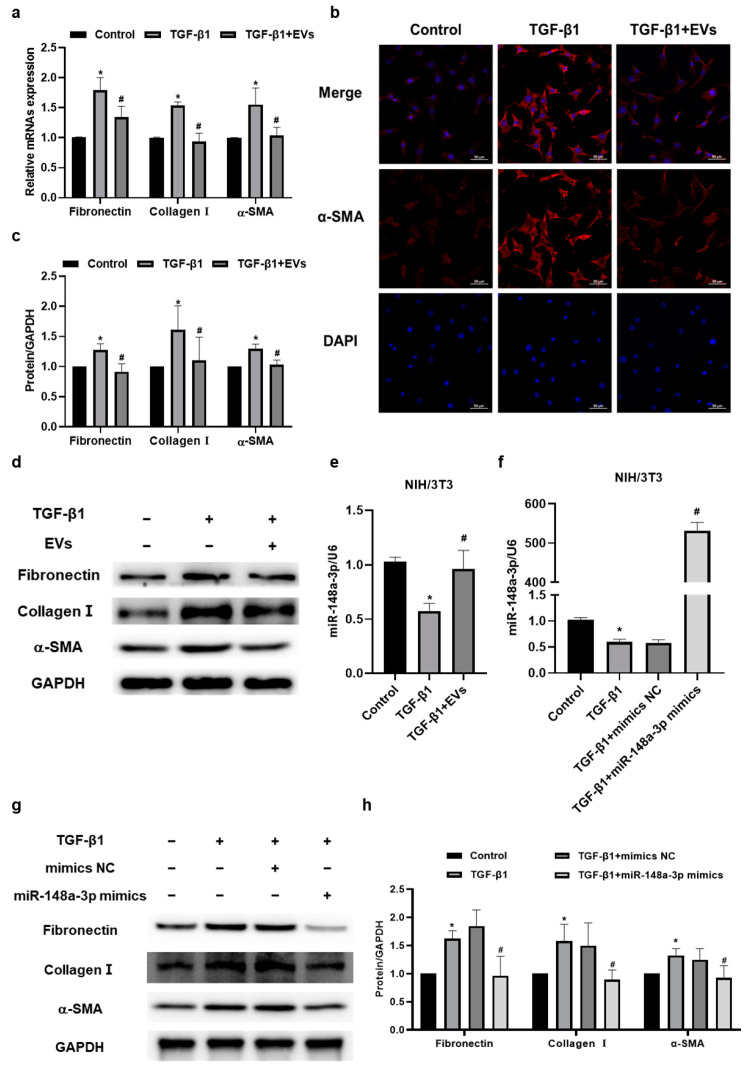
MiR-148a-3p in hucMSC-EVs suppressed TGF-β1-induced collagen deposition in NIH/3T3 cells. (**a**) qRT-PCR analysis of Fibronectin, Collagen I, and α-SMA mRNA expression changes in NIH/3T3 cells after hucMSC-EV intervention. (**b**) Immunofluorescence staining of α-SMA (red) levels in NIH/3T3 cells after hucMSC-EV intervention; cell nuclei stained with DAPI (blue). Scale bar: 50 μm. (**c**,**d**) Western blotting analysis of Fibronectin, Collagen I, and α-SMA protein expression changes in NIH/3T3 cells after hucMSC-EV intervention. (**e**) qRT-PCR analysis of miR-148a-3p expression changes in NIH/3T3 cells after hucMSC-EV intervention. * *p* < 0.05 compared with the control group; # *p* < 0.05 compared with the TGF-β1 group. (**f**) qRT-PCR analysis of miR-148a-3p in NIH/3T3 cells stimulated by TGF-β1 for 24 h after transfection with miR-148a-3p mimics or mimicsNC for 12 h. (**g**,**h**) Western blotting analysis of Fibronectin, Collagen I, and α-SMA in NIH/3T3 cells stimulated by TGF-β1 for 24 h after transfection with miR-148a-3p mimics or mimics NC for 12 h. * *p* < 0.05 compared with control group; # *p* < 0.05 compared with TGF-β1 + mimics NC group. Data are shown as the means ± SD (n ≥ 3).

To explore the specific biological processes that involve miR-148a-3p, we established a collagen secretion model by stimulating NIH/3T3 cells with the potent pro-fibrosis factor TGF-β1 (Figure S1a–c). EVs and their contents could be transferred between cells through various mechanisms. Our study found that PKH67-labeled hucMSC-EVs were localized in the perinuclear region of NIH/3T3 cells, indicating that they could enter the cytoplasm of NIH/3T3 cells (Figure S1d). Then, we found that the mRNA expressions of Fibronectin, Collagen I, and α-SMA were downregulated in the TGF-β1 + EVs group compared with the TGF-β1 group (Figure 4a), which was consistent with the protein levels detected via Western blotting (Figure 4c,d). Immunofluorescence staining further confirmed a decrease in α-SMA, a marker of myofibroblasts, in NIH/3T3 cells following hucMSC-EV intervention (Figure 4b). Moreover, hucMSC-EV intervention reversed the downregulation of miR-148a-3p in NIH/3T3 cells stimulated with TGF-β1, as shown in Figure 4e. These results suggested a potential association between miR-148a-3p and the inhibitory effect of hucMSC-EVs in TGF-β1-induced collagen deposition in NIH/3T3 cells. 

To further investigate the effect of miR-148a-3p in NIH/3T3 cells, miR-148a-3p mimics were constructed and transfected into the NIH/3T3 cells to artificially elevate its expression level (Figure S2). Subsequently, we examined the influence of miR-148a-3p on fibroblasts’ collagen synthesis and secretion by transfecting NIH/3T3 cells with miR-148a-3p mimic prior to TGF-β1 stimulation. The expression level of miR-148a-3p was remarkably upregulated following transfection with the miR-148a-3p mimics (Figure 4f). Furthermore, the results of our Western blotting analysis showed that the overexpression of miR-148a-3p could suppress the protein expression levels of Fibronectin, Collagen I, and α-SMA (Figure 4g,h), indicating the antifibrotic effect of miR-148a-3p in inhibiting fibroblast collagen synthesis and secretion. Taken together, it was demonstrated that miR-148a-3p in hucMSC-EVs could suppress TGF-β1-induced collagen deposition in NIH/3T3 cells. 

### 2.5. The Amelioratory Effect of hucMSC-EVs on Silica-Induced Pulmonary Fibrosis by Targeting Hsp90b1 *via* miR-148a-3p

**Figure 5:**
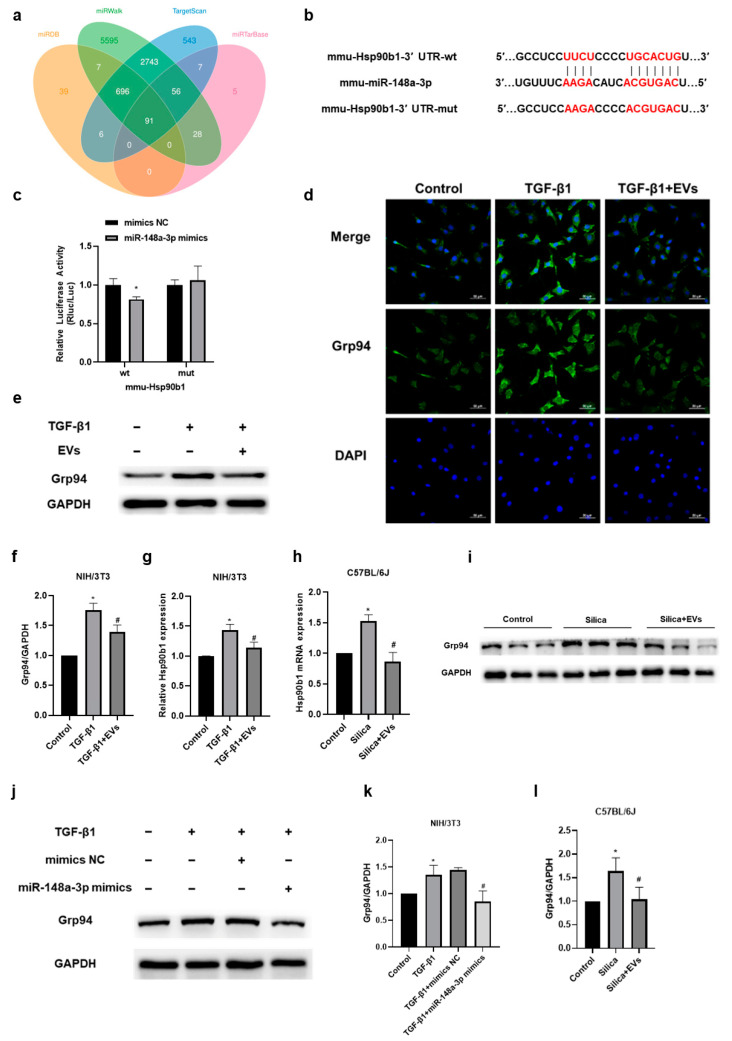
The amelioratory effect of hucMSC-EVs on silica-induced pulmonary fibrosis by targeting heat shock protein 90 beta family member 1 (Hsp90b1) via miR-148a-3p. (**a**) Venn map diagram illustrating target gene predictions. (**b**,**c**) Our dual-luciferase reporter assay verified Hsp90b1 as a target of miR-148a-3p. * *p* < 0.05 compared with the mimics NC group. (**d**–**f**) Immunofluorescence staining and Western blotting were used to detect the protein level of Grp94 (encoded by Hsp90b1) in the NIH/3T3 cells. (**g**) The gene expression of Hsp90b1 in the NIH/3T3 cells was quantified via qRT-PCR. * *p* < 0.05 compared with the control group; # *p* < 0.05 compared with the TGF-β1 group. (**h**) The gene level of Hsp90b1 in mice was detected via qRT-PCR. (**i**,**l**) Western blotting was employed to analyze the protein level of Grp94 in mice. * *p* < 0.05 compared with the control group; # *p* < 0.05 compared with the silica group. (**j**,**k**) Western blotting analysis of Grp94 in NIH/3T3 cells stimulated by TGF-β1 for 24 h after transfection with miR-148a-3p mimics or mimics NC for 12 h. * *p* < 0.05 compared with the control group; # *p* < 0.05 compared with the TGF-β1 + mimics NC group. Data are shown as the means ± SD (n ≥ 3).

To clarify the potential molecular mechanism of miR-148a-3p, we used various prediction algorithms, including TargetScan, miRDB, miRTarBase, and miRWalk (Figure 5a), to identify Hsp90b1 as a potential target of miR-148a-3p. To verify the interaction between miR-148a-3p and Hsp90b1, we performed a dual-luciferase reporter gene assay. The wild-type (wt) 3′-UTR sequence and the mutant (mut) 3′-UTR sequence of Hsp90b1 were cloned to construct reporter plasmids, respectively (Figure 5b). The results displayed that the co-transfection of miR-148a-3p mimics and the wt-Hsp90b1 reporter gene plasmid decreased the luciferase activity significantly, whereas no effect was observed with the mutant plasmid (Figure 5c), confirming that Hsp90b1 was a target of miR-148a-3p. We subsequently sought to determine whether Hsp90b1 was correlated with the inhibition of pulmonary fibrosis by hucMSC-EVs. Our in vivo results showed that Grp94, encoded by Hsp90b1 [32], was upregulated in the TGF-β1-stimulated NIH/3T3 cells but downregulated after hucMSC-EV treatment (Figure 5d–f), and the change at the gene level of Hsp90b1 was consistent with the protein level (Figure 5g). We further examined the mRNA and protein levels of Hsp90b1 in vivo. Our results showed that the gene and protein level of Hsp90b1 in mice lung tissue was increased upon silica exposure, while hucMSC-EV intervention could mitigate this upregulation (Figure 5h,i,l). Additionally, we observed that the overexpression of miR-148a-3p suppressed the upregulation of Grp94 induced by TGF-β1 in NIH/3T3 cells (Figure 5j,k). These observations collectively confirmed that miR-148a-3p might play an important role in hucMSC-EVs’ amelioration of silica-induced pulmonary fibrosis via affecting the function of fibroblasts by targeting Hsp90b1.

### 2.6. The Inhibition of Hsp90b1 Suppresses Fibrosis-Related Proteins in NIH/3T3 Cells

**Figure 6:**
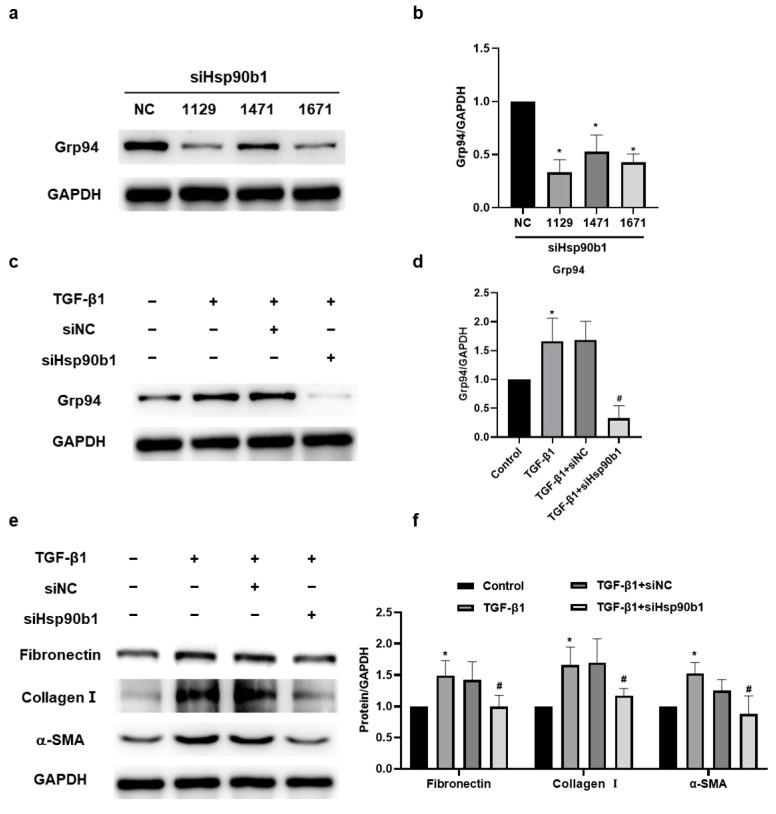
Inhibition of Hsp90b1 suppresses fibrosis-related proteins in NIH/3T3 cells. (**a**,**b**) Western blotting analysis of Grp94 in NIH/3T3 cells transfected with three small interfering RNAs (siRNAs) of Hsp90b1 (siHsp90b1-1129/1471/1671) and its negative Control (NC). * *p* < 0.05 compared with the siNC group. (**c**,**d**) Western blotting analysis of Grp94 in NIH/3T3 cells stimulated by TGF-β1 for 24 h after transfection with siHsp90b1-1129/NC for 12 h. (**e**,**f**) Western blotting analysis of Fibronectin, Collagen I, and α-SMA in NIH/3T3 cells stimulated by TGF-β1 for 24 h after transfection with siHsp90b1-1129/NC for 12 h.* *p* < 0.05 compared with the control group. # *p* < 0.05 compared with the TGF-β1 + siNC group. Data are shown as the means ± SD (n ≥ 3).

To determine the specific effect of Hsp90b1 in silica-induced pulmonary fibrosis, we designed three small interfering RNAs (siRNAs) (siHsp90b1-1129/1471/1671) targeting Hsp90b1 and assessed their efficacy. Among them, siHsp90b1-1129 exhibited the highest interference effect and was selected for transfection into NIH/3T3 (Figure 6a,b). Our Western blotting results showed that the siHsp90b1-1129 effectively decreased the Grp94 protein level in NIH/3T3 cells stimulated with TGF-β1 (Figure 6c,d). In addition, siHsp90b1 markedly attenuated the expressions of Fibronectin, Collagen I, and α-SMA in NIH/3T3 cells induced by TGF-β1, indicating that the suppressed collagen synthesis and secretion of fibroblasts might be attributed to the downregulation of Hsp90b1 (Figure 6e,f). These findings suggested that the downregulation of Hsp90b1 could inhibit the fibrosis-related proteins in NIH/3T3 cells.

## 3. Discussion

Evidence in the literature has demonstrated that MSC-EVs have the potential to antagonize silica-induced pulmonary fibrosis, and the mechanism is complex [33,34]. In the present study, we found that miR-148a-3p in hucMSC-EVs was associated with hucMSC-EVs in improving silica-induced pulmonary fibrosis and preliminarily identified that miR-148a-3p exerted an antifibrotic effect via inhibiting the collagen synthesis and secretion of fibroblasts by targeting Hsp90b1.

Since the paracrine function of MSCs is the key aspect of their therapeutic use, it has been suggested that mesenchymal stem cells should be re-named using “medicinal signaling cells” or “message secreting cells” to emphasize their role as storehouses that store and release biologically active substances with therapeutic effects [35]. As the main component of paracrine, EVs are wrapped with various cargos derived from parent cells, including miRNAs, mRNAs, proteins, and more. These EVs act as dynamic shuttles, facilitating the exchange of information and transfer of materials between cells. Through this process, EVs regulate the biological functions of target cells, contributing to intercellular communication. Studies have highlighted that the biological role of EVs can be partly attributed to miRNAs, one of the dominating effectors within the contents of EVs, particularly in regulating respiratory function [29,36]. In addition, studies have shown that an abnormal expression of miRNAs is closely related to silica-induced pulmonary fibrosis [31,37]. Hence, we sequenced miRNAs in hucMSC-EVs and MRC-5-EVs. HucMSCs were selected due to their inherent advantages of convenient isolation, abundant cell yield, and high gene transfection efficiency. These distinctive attributes make hucMSCs optimal candidates for cellular therapeutic applications [38,39,40]. MRC-5-EVs were employed as a control due to their shared fibroblast morphology, comparable surface markers, and differentiation ability [41]. Notably, our sequencing results showed that miR-148a-3p exhibited the highest abundance in hucMSC-EVs among the differentially expressed miRNAs.

MiR-148a-3p is a member of the miR-148/miR-152 family and includes three highly conserved miRNAs (miR-148a, miR-148b, and miR-152), which remain relatively unknown in fibrosis. Studies have discovered that miR-148a-3p is downregulated in liver fibrosis [42]. In a rat model of alcoholic liver fibrosis, miR-148a-3p inhibits the expression of fibrosis-related markers [23], and the use of miR-148a-3p mimics or inhibitors has the potential to reduce or enhance the activation of hepatic stellate cells and modulate the expression of collagen fibers [43,44]. However, the relationship between miR-148a-3p and silicosis remains unclear. An analysis of the public database GSE80555 provided evidence indicating that miR-148a-3p is downregulated in silicosis patients. In this study, we discovered that the expression of miR-148a-3p was downregulated in both silica-exposed mice and fibroblasts, and we subsequently found that miR-148a-3p mimics could inhibit the TGF-β1-induced collagen synthesis and secretion of fibroblasts. These intriguing findings sparked our interest in exploring the potential anti-fibrotic role of miR-148a-3p in silicosis. Our previous study demonstrated that miRNAs within hucMSC-EVs were involved in the inhibition of silica-induced pulmonary fibrosis in mice by hucMSC-EVs, including let-7i-5p and miR-26a-5p [20,21]. Given these findings, we supposed that miR-148a-3p might also play a significant role in the anti-fibrotic effects exerted by hucMSC-EVs.

To test our supposition, we conducted in vivo and in vitro studies. We found that hucMSC-EV intervention effectively ameliorated the pathological lung tissue injury induced by silica and reduced the expression levels of fibrosis-related proteins. On this basis, a significant upregulation of miR-148a-3p was also observed in the lung tissue of mice treated with hucMSC-EVs compared to the silica group. These intriguing results strongly support the hypothesis that miR-148a-3p might be one of the mediators of the inhibition of silica-induced lung fibrosis by hucMSC-EVs. We further explored the specific biological processes in which miR-148a-3p may be intricately involved. In previous studies, we found that miR-148a-3p was related to fibroblast function. Notably, during the progression of fibrosis, fibroblasts undergo activation triggered by various factors, thereby playing a key role in the synthesis and secretion of collagen, the remodeling of the ECM, and the promotion of fibrosis [5]. As a very well established and powerful pro-fibrosis factor, TGF-β1 is involved in proliferation, activation, and collagen synthesis and secretion by fibroblasts, which plays a crucial role in silicosis and other kinds of pulmonary fibrosis [45,46,47]. In vitro experimental models related to silica-induced fibrosis often employ it as a common approach [30,37], which we also utilized in our study. We observed that hucMSC-EV intervention effectively inhibited TGF-β1-induced upregulation in collagen synthesis and secretion by fibroblasts and reduced the protein level of the fibroblast activation marker α-SMA. Additionally, hucMSC-EVs demonstrated the ability to restore the reduced miR-148a-3p levels induced by TGF-β1 in fibroblasts. Moreover, we also observed that miR-148a-3p mimics effectively suppressed TGF-β1-induced fibroblast collagen synthesis and secretion. These findings suggest that hucMSC-EVs possess the potential to ameliorate silica-induced lung fibrosis through the anti-fibrotic action of miR-148a-3p. In other words, the anti-fibrotic function of hucMSC-EVs may, in part, be attributed to its miR-148a-3p content. Consistent with our results, many studies have demonstrated that MSC-EVs play an important role in coordinating the fibrotic disease process by regulating biological processes through the delivery of miRNAs. For instance, it was demonstrated that human placenta-derived MSC-EVs could reduce lung inflammation and fibrosis induced by radiation via transferring miR-214-3p [48]. Adipose-derived MSC-EVs delivered miR-181-5p and miR-126 to prevent liver fibrosis and myocardial infarction [49,50]. Additionally, in another study, bone marrow-derived MSC-EVs transmitted miR-23a-3p, and miR-182-5p reversed the progression of LPS-induced lung injury and fibrosis [51]. The protective effect of the bilayer membrane of EVs enables the efficient transport of miRNAs to target cells regardless of the distance [52]. This characteristic further underscores the potential of MSC-EVs as a promising drug delivery system [53].

Previous studies have suggested that MSC-EVs may profoundly alter the genetic manipulation within cells due to their cargo of miRNAs, which can regulate genetic expression at the post-transcriptional level by targeting specific mRNAs. We aimed to investigate the function of miR-148a-3p by identifying its target genes. Among these target genes, we focused on Hsp90b1, also known as Grp94, which is the endoplasmic reticulum subtype of Hsp90 [25]. Hsp90 is a class of highly conserved chaperone proteins and is one of the most abundant proteins in eukaryotic cells under non-stress conditions, accounting for about 1–2% of total protein content [54]. Hsp90 consists of four isoforms located in different cellular compartments, including the cytoplasm (Hsp90α, Hsp90β), endoplasmic reticulum (Hsp90b1), and mitochondria (TRAP1). Notably, studies have found that two cytoplasmic subtypes of Hsp90—Hsp90α and Hsp90β—are elevated in patients with idiopathic pulmonary fibrosis [55]. These isoforms have been shown to promote the progress of pulmonary fibrosis by promoting fibroblast activation, likely involving the TGF-β and PI3K/AKT pathway [56,57]. However, studies on the association between Hsp90b1 and fibrosis are rare. Hsp90b1 is a typical molecular chaperone in the endoplasmic reticulum that is involved in the folding and assembly of many proteins. In the past few years, studies have found that the expression of Hsp90b1 is upregulated in liver fibrosis, which is related to endoplasmic reticulum stress [28], and through proteomics and genetic investigations, Hsp90b1 has been identified as one of the biomarkers of hepatic fibrosis [27]. Our study found that Hsp90b1 was a target gene of miR-148a-3p, and Hsp90b1 was increased in the lung tissue of mice in the silica-exposed group and upregulated in the fibroblasts stimulated by TGF-β1. Remarkably, hucMSC-EV intervention effectively suppressed the expression of Hsp90b1 both in vivo and in vitro, indicating the involvement of Hsp90b1 in the mechanism underlying the inhibition of silica-induced pulmonary fibrosis by hucMSC-EVs. Furthermore, our results revealed that miR-148a-3p mimics inhibited the TGF-β1-induced elevation of Hsp90b1 in fibroblasts. Importantly, the downregulation of Hsp90b1 could inhibit the collagen synthesis and secretion of fibroblasts. Taken together, these results suggest that hucMSC-EVs exert their inhibitory effects on fibroblast collagen synthesis and secretion and silica-induced pulmonary fibrosis in mice through the targeted delivery of miR-148a-3p, which downregulates Hsp90b1. However, the additional mechanism of Hsp90b1-regulated downstream factors is not discussed in this paper; we aim to explore this in the future.

## 4. Materials and Methods

### 4.1. HucMSCs Culture and hucMSC-EV Isolation and Identification

HucMSCs and medium were purchased from NUWACELL Co., Ltd. (Anhui, China). A 3D FloTrix miniSpin bioreactor (Beijing CytoNiche Biotechnology Co., Ltd., Beijing, China) was used for the 3D dynamic culture of hucMSCs, which were placed into the bioreactor at 37 °C in a humidified atmosphere with 5% CO_2_. 

The hucMSCs culture medium was collected before being centrifuged at 2000× *g* for 10 min and then at 10,000× *g* for 30 min. The supernatant was subjected to a 0.22 μm filter membrane (Millipore, Billerica, MA, USA), followed by a 100 kDa centrifugal filter device (Millipore, Billerica, MA, USA) and centrifuged at 4000× *g* for 20 min and then at 100,000× *g* for 70 min. All centrifugation steps were performed at 4 °C.

The hucMSC-EVs were identified by using Transmission Electron Microscopy (TEM) (Hitachi HT-7700, Tokyo, Japan), Nanoparticle Tracking Analysis (ZetaView PMX 110, Particle Metri, Meerbusch, Germany), and Western blotting analysis. Antibodies and dilution ratios are available in Supplementary Materials Table S1.

### 4.2. Animal Models

A total of 60 male C57BL/6J mice (20–22 g) were purchased from Vital River Laboratory Animal Technology (Beijing, China). The mice were randomly divided into three groups (n = 20 in each group): the control group, the silica group, and the silica + hucMSC-EVs group. The mice were anesthetized with 350 mg/kg of tribromoethanol (Sigma, St. Louis, MI, USA). The mice were treated with intratracheal silica suspension (2.5 mg/100 μL), except for the control group, which received the same volume of saline. In the silica + hucMSC-EVs group, hucMSC-EVs (200 μg/100 μL) were injected intravenously every four days at day 1 post silica instillation. The mice in the control and silica groups were injected with an equal volume of saline. The mice in these groups were sacrificed on the 30th day of the experiment, and lung tissue sections were collected for examination. The detailed operation steps have been described previously [20]. This study was approved by the Laboratory Animal Care and Use Committee at Capital Medical University in Beijing, China (AEEI-2018-223).

### 4.3. Histopathology

The lung tissues of mice were soaked in 10% formalin and then embedded in paraffin to make paraffin sections. HE and Masson staining were used for assessment. Section observation was performed using the digital slide scanner, Pannoramic Scan (3DHISTECH, Ltd., Budapest, Hungary).

### 4.4. NIH/3T3 Cell Culture and Treatment

NIH/3T3 cells (murine fibroblast cell line) were purchased from the Cell Resource of the Chinese Academy of Sciences (Beijing, China) and cultured with DMEM (Corning, Manassas, VA, USA) and 10% fetal bovine serum (FBS) (Gibco, Auckland, New Zealand) at 37 °C with 5% humidified CO_2_. The NIH/3T3 cells were treated with 5 ng/mL recombinant TGF-β1 (PeproTech, Cranbury, NJ, USA) and 50 μg/mL hucMSC-EVs for 24 h.

MiR-148a-3p mimics, siRNA-Hsp90b1, and each corresponding negative control (NC) were synthesized by Sangong Biotech Co., Ltd. (Shanghai, China). Lipofectamine™ RNAiMAX Transfection Reagent (Thermo Fisher Scientific, Carlsbad, CA, USA) was used for cell transfection according to the manufacturer’s instructions. At 12 h after transfection, the NIH/3T3 cells were treated with 5 ng/mL TGF-β1 for 24 h. The sequences are shown in Supplementary Materials Table S2.

### 4.5. In Vitro Tracking

HucMSC-EVs were labeled with PHK67 dye (Umibio Co., Ltd., Shanghai, China) and incubated with NIH/3T3 cells for 24 h, after which excess dye was washed off with DPBS (Corning, Manassas, VA, USA), incubated with Hoechst 33342 for 10 min (Invitrogen, Eugene, OR, USA) to label the cell nuclei, and photographed using laser-scanning confocal fluorescence microscopy (ECLIPSE Ti2, Nikon, Tokyo, Japan).

### 4.6. Western Blotting Analysis

The proteins of lung tissues and cells were extracted by using Radioimmunoprecipitation (RIPA) lysis buffer (Beyotime Biotechnology, Shanghai, China) and quantified by using the BCA Protein Assay Kit (Beijing Dinguo Changsheng Biotechnology Co., Ltd., Beijing, China). The protein samples were separated on SDS-PAGE and transferred onto PVDF membranes (Millipore, Billerica, MA, USA). Subsequently, it was blocked in 5% Difco^TM^ Skim Milk (BD Difco, Sparks, MD, USA) and incubated overnight at 4 °C with primary antibodies. The blots were visualized using horse radish peroxidase (HRP)-conjugated secondary antibodies and the ECL Detection Reagent (NCM Biotech, Suzhou, China) and were imaged using the Tanon-5200 system (Beijing Yuan Ping Hao Biotech, Shanghai, China). The densities of the bands were quantified using Image J software (1.52a;Java 1.8.0_112[64-bit]). Antibodies and dilution ratios are available in Supplementary Materials Table S1.

### 4.7. RNA Isolation and Quantitative Real-Time PCR (qRT-PCR) Analysis

The total RNA was isolated by using TRIzol reagent (Thermo Fisher Scientific, USA). The reverse transcription of mRNA into cDNA was performed using the TransScript First-Strand cDNA Synthesis SuperMix (AT301, TransGen Biotech, Beijing, China) and using the TransScript miRNA FirstStrand cDNA Synthesis SuperMix (AT351, TransGen Biotech, Beijing, China). qRT-PCR was performed using the CFX96 real-time qPCR detection system (Bio-Rad, Hercules, CA, USA) using the PerfectStart Green qPCR SuperMix (AQ601, TransGen Biotech, Beijing, China). The relative expression among groups was calculated using the 2^−ΔΔCt^ method. The primer sequences are available in Supplementary Materials Table S3.

### 4.8. Immunofluorescence

The NIH/3T3 cell samples were fixed with 4% paraformaldehyde, permeabilized with 0.3% Triton X-100, blocked with 1% BSA, and incubated with the primary antibodies at 4 °C overnight. Fluorescently labeled secondary antibodies were used for incubation for 1 h at room temperature before staining the nuclei with DAPI (ZLI-9557, ZSGB-BIO, Beijing, China). Images were taken using laser-scanning confocal fluorescence microscopy (ECLIPSE Ti2, Nikon, Japan). Antibodies and dilution ratios are available in Supplementary Materials Table S4.

### 4.9. Dual-Luciferase Reporter Gene Assay

Wild-type and mutant Hsp90b1 3′-UTR dual-luciferase plasmids (Guangzhou RiboBio Co., Ltd., Guangzhou, China) were designed and synthesized. The plasmids were transferred with miR-148a-3p mimics/NC into 293T cells using Lipofectamine™ 6000 (Beyotime, Shanghai, China). Following 48 h of transfection, the luciferase activity was assessed using a dual-luciferase assay (Promega, Madison, WI, USA).

### 4.10. Statistical Analysis

All experiments were repeated at least three times independently. The Statistical Package for Social Sciences version 24.0 (SPSS Inc., Chicago, IL, USA) was used for our statistical analysis. The Shapiro-Wilk test was applied to test the normality of the data, and all normally distributed data are presented as mean ± standard deviation (SD). The Levene test was used to test the homogeneity of variance. Student’s *t*-test was used to facilitate a comparison between the two groups. A one-way analysis of variance (ANOVA) was used to compare between multiple groups, and this was followed by a multiple comparison test using a LSD test or Tamhane test. *p* < 0.05 was set as the level for statistical significance.

## 5. Conclusions

In summary, our study primarily demonstrates that the presence of abundant miR-148a-3p within hucMSC-EVs mediated their capability of attenuating silica-induced pulmonary fibrosis in mice. Furthermore, we have identified a potential mechanism whereby miR-148a-3p exerts its anti-fibrotic effects by suppressing collagen synthesis and secretion in fibroblasts through the targeted inhibition of Hsp90b1. These findings provide a promising theoretical foundation in the clinical application of hucMSC-EVs for the treatment of silicosis.

## Data Availability

Please contact the corresponding author for data requests.

## Supplementary Materials

The following supporting information can be downloaded at: https://www.mdpi.com/article/10.3390/ijms241914477/s1.

## Abbreviations

α-SMA	alpha-smooth muscle actin
ECM	extracellular matrix
EVs	extracellular vesicles
FBS	fetal bovine serum
HE	hematoxylin and eosin
HRP	horse radish peroxidase
Hsp90	heat shock protein 90
Hsp90b1	heat shock protein 90 beta family member 1
HucMSCs	human umbilical cord mesenchymal stem cells
HucMSC-EVs	human umbilical cord mesenchymal stem cell-derived extracellular vesicles
MSCs	mesenchymal stem cells
MSC-EVs	mesenchymal stem cell-derived extracellular vesicles
MiRNA	microRNA
MRC-5-EVs	human embryonic lung fibroblast-derived extracellular vesicles
Mut	mutant
NIH/3T3 cell	murine fibroblast cell line
NTA	Nanoparticle Tracking Analysis
qRT-PCR	quantitative real-time polymerase chain reaction
SiRNAs	small interfering RNAs
TEM	Transmission Electron Microscope
TGF-β1	transforming growth factor-β1
Wt	wild-type
3D	three-dimensional
3′ UTR	3′ untranslated region

## References

[1] Leung C.C., Yu I.T., Chen W. (2012). Silicosis. Lancet.

[2] Hao X., Jin Y., Zhang Y., Li S., Cui J., He H., Guo L., Yang F., Liu H. (2023). Inhibition of Oncogenic Src Ameliorates Silica-Induced Pulmonary Fibrosis via PI3K/AKT Pathway. Int. J. Mol. Sci..

[3] Lian X., Chen X., Sun J., An G., Li X., Wang Y., Niu P., Zhu Z., Tian L. (2017). MicroRNA-29b inhibits supernatants from silica-treated macrophages from inducing extracellular matrix synthesis in lung fibroblasts. Toxicol. Res..

[4] Chanda D., Otoupalova E., Smith S.R., Volckaert T., De Langhe S.P., Thannickal V.J. (2019). Developmental pathways in the pathogenesis of lung fibrosis. Mol. Asp. Med..

[5] Pakshir P., Hinz B. (2018). The big five in fibrosis: Macrophages, myofibroblasts, matrix, mechanics, and miscommunication. Matrix Biol..

[6] Calabrese F., Lunardi F., Tauro V., Pezzuto F., Fortarezza F., Vedovelli L., Faccioli E., Balestro E., Schiavon M., Esposito G. (2022). RNA Sequencing of Epithelial Cell/Fibroblastic Foci Sandwich in Idiopathic Pulmonary Fibrosis: New Insights on the Signaling Pathway. Int. J. Mol. Sci..

[7] Cheng D., Wang Y., Li Z., Xiong H., Sun W., Xi S., Zhou S., Liu Y., Ni C. (2022). Liposomal UHRF1 siRNA shows lung fibrosis treatment potential through regulation of fibroblast activation. JCI Insight.

[8] Richeldi L., Collard H.R., Jones M.G. (2017). Idiopathic pulmonary fibrosis. Lancet.

[9] Kardia E., Zakaria N., Sarmiza Abdul Halim N.S., Widera D., Yahaya B.H. (2017). The use of mesenchymal stromal cells in treatment of lung disorders. Regen. Med..

[10] Geiger S., Hirsch D., Hermann F.G. (2017). Cell therapy for lung disease. Eur. Respir. Rev..

[11] Harrell C.R., Sadikot R., Pascual J., Fellabaum C., Jankovic M.G., Jovicic N., Djonov V., Arsenijevic N., Volarevic V. (2019). Mesenchymal Stem Cell-Based Therapy of Inflammatory Lung Diseases: Current Understanding and Future Perspectives. Stem Cells Int..

[12] Konala V.B., Mamidi M.K., Bhonde R., Das A.K., Pochampally R., Pal R. (2016). The current landscape of the mesenchymal stromal cell secretome: A new paradigm for cell-free regeneration. Cytotherapy.

[13] Han Y., Yang J., Fang J., Zhou Y., Candi E., Wang J., Hua D., Shao C., Shi Y. (2022). The secretion profile of mesenchymal stem cells and potential applications in treating human diseases. Signal Transduct. Target Ther..

[14] Keshtkar S., Azarpira N., Ghahremani M.H. (2018). Mesenchymal stem cell-derived extracellular vesicles: Novel frontiers in regenerative medicine. Stem Cell Res. Ther..

[15] Vizoso F.J., Eiro N., Cid S., Schneider J., Perez-Fernandez R. (2017). Mesenchymal Stem Cell Secretome: Toward Cell-Free Therapeutic Strategies in Regenerative Medicine. Int. J. Mol. Sci..

[16] Diener C., Keller A., Meese E. (2022). Emerging concepts of miRNA therapeutics: From cells to clinic. Trends Genet..

[17] Kilikevicius A., Meister G., Corey D.R. (2022). Reexamining assumptions about miRNA-guided gene silencing. Nucleic Acids Res..

[18] Wan X., Chen S., Fang Y., Zuo W., Cui J., Xie S. (2020). Mesenchymal stem cell-derived extracellular vesicles suppress the fibroblast proliferation by downregulating FZD6 expression in fibroblasts via micrRNA-29b-3p in idiopathic pulmonary fibrosis. J. Cell Physiol..

[19] Kadota T., Fujita Y., Araya J., Watanabe N., Fujimoto S., Kawamoto H., Minagawa S., Hara H., Ohtsuka T., Yamamoto Y. (2021). Human bronchial epithelial cell-derived extracellular vesicle therapy for pulmonary fibrosis via inhibition of TGF-β-WNT crosstalk. J. Extracell. Vesicles.

[20] Xu C., Hou L., Zhao J., Wang Y., Jiang F., Jiang Q., Zhu Z., Tian L. (2022). Exosomal let-7i-5p from three-dimensional cultured human umbilical cord mesenchymal stem cells inhibits fibroblast activation in silicosis through targeting TGFBR1. Ecotoxicol. Environ. Saf..

[21] Zhao J., Jiang Q., Xu C., Jia Q., Wang H., Xue W., Wang Y., Zhu Z., Tian L. (2023). MiR-26a-5p from HucMSC-derived extracellular vesicles inhibits epithelial mesenchymal transition by targeting Adam17 in silica-induced lung fibrosis. Ecotoxicol. Environ. Saf..

[22] Tian S., Zhou X., Zhang M., Cui L., Li B., Liu Y., Su R., Sun K., Hu Y., Yang F. (2022). Mesenchymal stem cell-derived exosomes protect against liver fibrosis via delivering miR-148a to target KLF6/STAT3 pathway in macrophages. Stem Cell Res. Ther..

[23] Xiong J., Ni J., Chen C., Wang K. (2020). miR-148a-3p regulates alcoholic liver fibrosis through targeting ERBB3. Int. J. Mol. Med..

[24] Woo S.J., Kim Y., Jung H., Lee J.J., Hong J.Y. (2022). MicroRNA 148a Suppresses Tuberculous Fibrosis by Targeting NOX4 and POLDIP2. Int. J. Mol. Sci..

[25] Chen B., Piel W.H., Gui L., Bruford E., Monteiro A. (2005). The HSP90 family of genes in the human genome: Insights into their divergence and evolution. Genomics.

[26] Cabaud-Gibouin V., Durand M., Quéré R., Girodon F., Garrido C., Jego G. (2023). Heat-Shock Proteins in Leukemia and Lymphoma: Multitargets for Innovative Therapeutic Approaches. Cancers.

[27] Husain H., Waseem M., Ahmad R. (2021). Proteomic and molecular evidences of Il1rl2, Ric8a, Krt18 and Hsp90b1 modulation during experimental hepatic fibrosis and pomegranate supplementation. Int. J. Biol. Macromol..

[28] San-Miguel B., Crespo I., Sánchez D.I., González-Fernández B., Ortiz de Urbina J.J., Tuñón M.J., González-Gallego J. (2015). Melatonin inhibits autophagy and endoplasmic reticulum stress in mice with carbon tetrachloride-induced fibrosis. J. Pineal. Res..

[29] Li S., Zhang J., Feng G., Jiang L., Chen Z., Xin W., Zhang X. (2022). The Emerging Role of Extracellular Vesicles from Mesenchymal Stem Cells and Macrophages in Pulmonary Fibrosis: Insights into miRNA Delivery. Pharmaceuticals.

[30] Sun W., Li Y., Ma D., Liu Y., Xu Q., Cheng D., Li G., Ni C. (2022). ALKBH5 promotes lung fibroblast activation and silica-induced pulmonary fibrosis through miR-320a-3p and FOXM1. Cell. Mol. Biol. Lett..

[31] Yuan J., Li P., Pan H., Xu Q., Xu T., Li Y., Wei D., Mo Y., Zhang Q., Chen J. (2021). miR-770-5p inhibits the activation of pulmonary fibroblasts and silica-induced pulmonary fibrosis through targeting TGFBR1. Ecotoxicol. Environ. Saf..

[32] Liu B., Staron M., Hong F., Wu B.X., Sun S., Morales C., Crosson C.E., Tomlinson S., Kim I., Wu D. (2013). Essential roles of grp94 in gut homeostasis via chaperoning canonical Wnt pathway. Proc. Natl. Acad. Sci. USA.

[33] Zhang E., Geng X., Shan S., Li P., Li S., Li W., Yu M., Peng C., Wang S., Shao H. (2021). Exosomes derived from bone marrow mesenchymal stem cells reverse epithelial-mesenchymal transition potentially via attenuating Wnt/β-catenin signaling to alleviate silica-induced pulmonary fibrosis. Toxicol. Mech. Methods.

[34] Bandeira E., Oliveira H., Silva J.D., Menna-Barreto R.F.S., Takyia C.M., Suk J.S., Witwer K.W., Paulaitis M.E., Hanes J., Rocco P.R.M. (2018). Therapeutic effects of adipose-tissue-derived mesenchymal stromal cells and their extracellular vesicles in experimental silicosis. Respir. Res..

[35] Caplan A.I. (2017). Mesenchymal Stem Cells: Time to Change the Name!. Stem Cells Transl. Med..

[36] Wang M.Y., Zhou T.Y., Zhang Z.D., Liu H.Y., Zheng Z.Y., Xie H.Q. (2021). Current therapeutic strategies for respiratory diseases using mesenchymal stem cells. MedComm.

[37] Sun J., Li Q., Lian X., Zhu Z., Chen X., Pei W., Li S., Abbas A., Wang Y., Tian L. (2019). MicroRNA-29b Mediates Lung Mesenchymal-Epithelial Transition and Prevents Lung Fibrosis in the Silicosis Model. Mol. Ther. Nucleic Acids.

[38] Ding D.C., Chang Y.H., Shyu W.C., Lin S.Z. (2015). Human umbilical cord mesenchymal stem cells: A new era for stem cell therapy. Cell Transpl..

[39] Hoffmann A., Floerkemeier T., Melzer C., Hass R. (2017). Comparison of in vitro-cultivation of human mesenchymal stroma/stem cells derived from bone marrow and umbilical cord. J. Tissue Eng. Regen. Med..

[40] Yin S., Ji C., Wu P., Jin C., Qian H. (2019). Human umbilical cord mesenchymal stem cells and exosomes: Bioactive ways of tissue injury repair. Am. J. Transl. Res..

[41] Zhang K., Na T., Wang L., Gao Q., Yin W., Wang J., Yuan B.Z. (2014). Human diploid MRC-5 cells exhibit several critical properties of human umbilical cord-derived mesenchymal stem cells. Vaccine.

[42] Chen W., Zhao W., Yang A., Xu A., Wang H., Cong M., Liu T., Wang P., You H. (2017). Integrated analysis of microRNA and gene expression profiles reveals a functional regulatory module associated with liver fibrosis. Gene.

[43] Huang W., Huang F., Zhang R., Luo H. (2021). LncRNA Neat1 expedites the progression of liver fibrosis in mice through targeting miR-148a-3p and miR-22-3p to upregulate Cyth3. Cell Cycle.

[44] Zhu J., Luo Z., Pan Y., Zheng W., Li W., Zhang Z., Xiong P., Xu D., Du M., Wang B. (2019). H19/miR-148a/USP4 axis facilitates liver fibrosis by enhancing TGF-β signaling in both hepatic stellate cells and hepatocytes. J. Cell. Physiol..

[45] Zhang Z.Q., Tian H.T., Liu H., Xie R. (2021). The role of macrophage-derived TGF-β1 on SiO(2)-induced pulmonary fibrosis: A review. Toxicol. Ind. Health.

[46] Rocha-Parise M., Santos L.M., Damoiseaux J.G., Bagatin E., Lido A.V., Torello C.O., Cohen Tervaert J.W., Queiroz M.L. (2014). Lymphocyte activation in silica-exposed workers. Int. J. Hyg. Environ. Health.

[47] Park S.J., Hahn H.J., Oh S.R., Lee H.J. (2023). Theophylline Attenuates BLM-Induced Pulmonary Fibrosis by Inhibiting Th17 Differentiation. Int. J. Mol. Sci..

[48] Lei X., He N., Zhu L., Zhou M., Zhang K., Wang C., Huang H., Chen S., Li Y., Liu Q. (2021). Mesenchymal Stem Cell-Derived Extracellular Vesicles Attenuate Radiation-Induced Lung Injury via miRNA-214-3p. Antioxid. Redox Signal.

[49] Qu Y., Zhang Q., Cai X., Li F., Ma Z., Xu M., Lu L. (2017). Exosomes derived from miR-181-5p-modified adipose-derived mesenchymal stem cells prevent liver fibrosis via autophagy activation. J. Cell Mol. Med..

[50] Luo Q., Guo D., Liu G., Chen G., Hang M., Jin M. (2017). Exosomes from MiR-126-Overexpressing Adscs Are Therapeutic in Relieving Acute Myocardial Ischaemic Injury. Cell Physiol. Biochem..

[51] Xiao K., He W., Guan W., Hou F., Yan P., Xu J., Zhou T., Liu Y., Xie L. (2020). Mesenchymal stem cells reverse EMT process through blocking the activation of NF-κB and Hedgehog pathways in LPS-induced acute lung injury. Cell Death Dis..

[52] Tkach M., Théry C. (2016). Communication by Extracellular Vesicles: Where We Are and Where We Need to Go. Cell.

[53] Crivelli B., Chlapanidas T., Perteghella S., Lucarelli E., Pascucci L., Brini A.T., Ferrero I., Marazzi M., Pessina A., Torre M.L. (2017). Mesenchymal stem/stromal cell extracellular vesicles: From active principle to next generation drug delivery system. J. Control. Release.

[54] Sreedhar A.S., Kalmár E., Csermely P., Shen Y.F. (2004). Hsp90 isoforms: Functions, expression and clinical importance. FEBS Lett..

[55] Korfei M., Schmitt S., Ruppert C., Henneke I., Markart P., Loeh B., Mahavadi P., Wygrecka M., Klepetko W., Fink L. (2011). Comparative proteomic analysis of lung tissue from patients with idiopathic pulmonary fibrosis (IPF) and lung transplant donor lungs. J. Proteome Res..

[56] Bellaye P.S., Shimbori C., Yanagihara T., Carlson D.A., Hughes P., Upagupta C., Sato S., Wheildon N., Haystead T., Ask K. (2018). Synergistic role of HSP90α and HSP90β to promote myofibroblast persistence in lung fibrosis. Eur. Respir. J..

[57] Sibinska Z., Tian X., Korfei M., Kojonazarov B., Kolb J.S., Klepetko W., Kosanovic D., Wygrecka M., Ghofrani H.A., Weissmann N. (2017). Amplified canonical transforming growth factor-β signalling via heat shock protein 90 in pulmonary fibrosis. Eur. Respir. J..

