# Supplementary material for: MiR-148a-3p within HucMSC-Derived Extracellular Vesicles Suppresses Hsp90b1 to Prevent Fibroblast Collagen Synthesis and Secretion in Silica-Induced Pulmonary Fibrosis
Source: Int J Mol Sci. 2023 Sep 23;24(19):14477. doi: 10.3390/ijms241914477 (PMC10572270; doi:10.3390/ijms241914477)
Supplement: Supplementary file 1 [file ijms-24-14477-s001.zip › ijms-2610917-supplementary.pdf]

Supplementary Material Figures

Figure S1: NIH/3T3 cells were stimulated by TGF-β1 to establish a collagen secretion model.

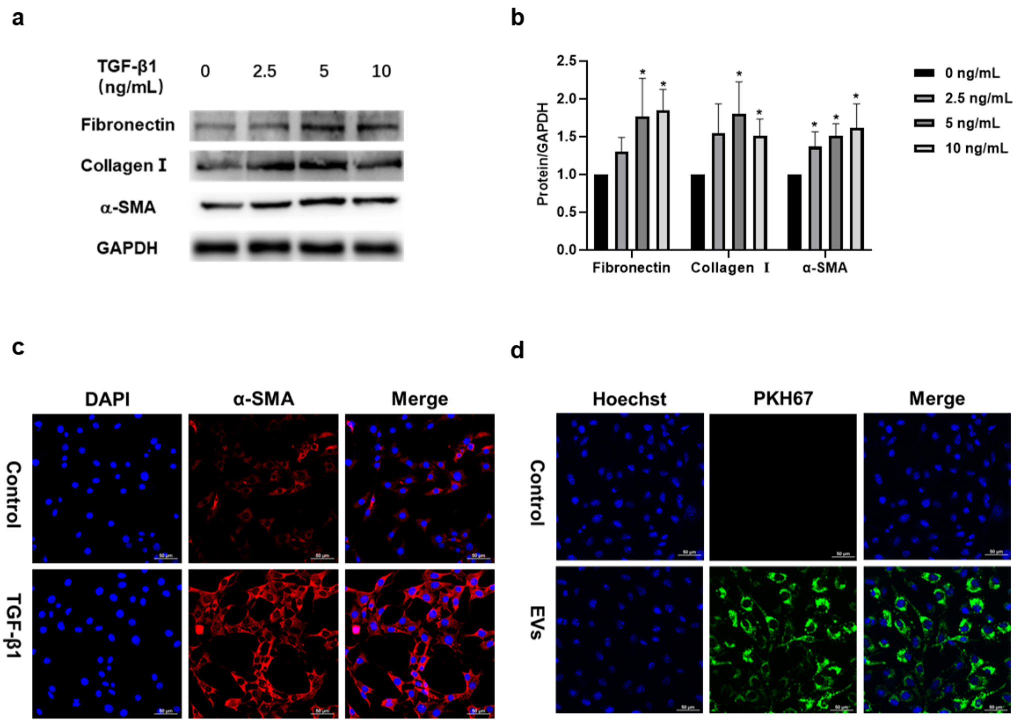

Figure S1. a, **b** NIH/3T3 cells were stimulated by 0, 2.5, 5, and 10 ng/mL TGF-β1 for 24 h, and protein levels of Fibronectin, Collagen I, and α-SMA were examined via Western blotting. **c** Immunofluorescence staining detected α-SMA (red) levels in NIH/3T3 cells, which were treated with 5 ng/mL TGF-β1 for 24h; DNA staining by DAPI (blue) represents cell nucleus; Scale bar: 50 μm. **d** Tracking hucMSC-EVs in NIH/3T3 cells; PKH67 green fluorescent dye was used to label the hucMSC-EVs. Hoechst blue fluorescent dye was applied to label the cell nucleus; Scale bar: 50 μm.

Figure S2: The transfection efficiency of miR-148a -3p mimics.

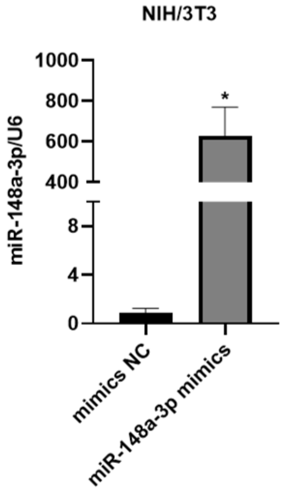

Figure S2. qRT-PCR analysis of miR-148a-3p in NIH/3T3 cells transfected with miR-148a-3p mimics and its negative Control (NC). * $p < 0.05$ compared with the mimics NC group. Data are shown as the means ± SD (n ≥ 3).

Supplementary Material Tables

Table S1: Antibodies for Western blotting.

Antibody	Manufacturer	Catalog	Dilution
CD81	Abcam	ab33697	1:1000
CD63	proteintech	25682-1-AP	1:1000
TSG101	Abcam	ab125011,	1:1000
Calnexin	Abcam	ab92573	1:1000
Fibronectin	Santa Cruz	sc-8422	1:300
Collagen I	Santa Cruz	sc-293182	1:300
α-SMA	Abcam	ab7817	1:3000
Grp94	Santa Cruz	sc-393492	1:800
GAPDH	CST	2118s	1:1000
Anti-mouse IgG,HRPlinked	CST	7076	1:5000
Anti-rabbitIgG,HRPlinked	CST	7074	1:5000

Table S2: The sequences of miR-148a-3p mimics/NC and siRNA-Hsp90b1.

Name		Sequences(5′-3′)
MiR-148a-3p mimics	sense	UCAGUGCACUACAGAACUUUGU
	antisense	AAAGUUCUGUAGUGCACUGAUU
Mimics NC	sense	UUGUACUACACAAAAGUACUG
	antisense	GUACUUUUGUGUAGUACAAUU
siHsp90b1-1471	sense	GGAAGAAGCUUGUCCGAAATT
	antisense	UUUCGGACAAGCUUCUUCCTT
siHsp90b1-1129	sense	CCAAAGAAGUAGAAGAAGATT
	Antisense	UCUUCUUCUACUUCUUUGGTT
siHsp90b1-1671	sense	GUGGAAAGAUGAAGGAAATT
	antisense	UUUCCUUCAUUCUUUCCACTT
siNC	sense	UUCUCCGAACGUGUCACGUTT
	antisense	ACGUGACACGUUCGGAGAATT

Table S3: The sequences used in qRT-PCR.

Name	Species	Sequences(5′-3′)	
miR-148a-3p	Human/Mouse	sense	CGTCAGTGCACTACAGAACTTTGT
miR-199a-3p	Human/Mouse	sense	CGCACAGTAGTCTGCACATTGGTTA
miR-143-3p	Human/Mouse	sense	CGCTGAGATGAAGCACTGTAGCTC
let-7i-5p	Human/Mouse	sense	CGCTGAGGTAGTAGTTTGTGCTGTT
miR-381-3p	Human/Mouse	sense	CCTATACAAGGGCAAGCTCTCTGT
miR-493-5p	Human/Mouse	sense	GCGCTTGTACATGGTAGGCTTTCATT
cel-miR-39-3p	Elegans	sense	TCTCACCGGGTGTAAATCAGCTTG
U6	Human/Mouse	sense	GACACGCAAATTCGTGAAGC
Fibronectin	Mouse	sense	CTATAGGATTGGAGACACGTGG
		antisense	CTGAAGCACTTTGTAGAGCATG
α-SMA	Mouse	sense	GAGCATCCGACACTGCTGAC
		antisense	GCACAGCCTGAATAGCCACA
Collagen I	Mouse	sense	TGAACGTGGTGTACAAGGTC
		antisense	CCATCTTTACCAGGAGAACCAT
Hsp90b1	Mouse	sense	CTTCTACCAGACACCAAGGCGTATG
		antisense	GTTCTTCCTCCACCTGTGCTTCAG
GAPDH	Mouse	sense	AAGAAGGTGGTGAAGCAGGC
		antisense	TCCACCACCCTGTTGCTGTA

Table S4: Antibodies for immunofluorescence.

Antibody	Manufacturer	Catalog	Dilution
α-SMA	Abcam	ab7817	1:150
Grp94	Abcam	ab3674	1:200
Anti-mouse IgG (H+L)	CST	8890	1:1000
Anti-rabbit IgG (H+L)	CST	4412	1:1000
